# Development of a Hop Functional Analog Derived from a Global Agrofood By-Product: Roasted Coffee Silverskin

**DOI:** 10.3390/molecules31071099

**Published:** 2026-03-27

**Authors:** Nadia Guzińska, Maria Dolores del Castillo, Edyta Kordialik-Bogacka

**Affiliations:** 1 Interdisciplinary Doctoral School, Lodz University of Technology, 116 Stefana Żeromskiego Street Lodz, 90-543 Lodz, Poland; 2 Institute of Fermentation Technology and Microbiology, Faculty of Biotechnology and Food Sciences, Lodz University of Technology, 171/173 Wólczańska, 90-530 Lodz, Poland; edyta.kordialik-bogacka@p.lodz.pl; 3 Instituto de Investigación en Ciencias de la Alimentación (CIAL) (CSIC-UAM), Campus de la Universidad Autónoma de Madrid, C/Nicolás Cabrera, 9, 28049 Madrid, Spain; mdolores.delcastillo@csic.es

**Keywords:** non-alcoholic beer, hop alternative, coffee by-product

## Abstract

Roasted coffee silverskin (RCSS) is a by-product of coffee production characterized by its content of phenolic compounds, including those contributing to bitterness. The aim of this study was to evaluate RCSS as an analog for hops in the production of non-alcoholic beer. Beers were developed using hops, RCSS, or a combination of both. Their sensory and physicochemical properties were evaluated, including bitterness, total phenolic content, and antioxidant capacity. Compared to hopped beer, the RCSS beer exhibited a significantly higher original gravity (7.11°P vs. 6.70°P), apparent extract (6.52°P vs. 6.20°P), and darker color (18.02 vs. 4.65 EBC). The total phenolic content was also significantly higher in the RCSS beer, reaching 0.51 ± 0.03 mg CGA/mL, which represents a 34% increase compared to the hopped variant. Importantly, the addition of RCSS had no negative effect on fermentation process. Moreover, the RCSS beer was characterized by improved overall sensory quality. These results indicate that RCSS is an innovative, sustainable alternative to hops, enhancing both sensory and functional properties while supporting zero-waste brewing strategies.

## 1. Introduction

Beer is one of the most widely consumed fermented beverages worldwide, whose sensory and functional properties arise from interactions between raw materials and biochemical transformations occurring during fermentation and maturation [1]. As a result, beer represents a complex biochemical matrix rich in carbohydrates, proteins, vitamins, minerals, polyphenols, and volatile compounds that collectively determine its quality and stability [2]. In recent years, the global market for non-alcoholic beverages has expanded rapidly, driven by increasing health awareness and changing consumer lifestyles. Non-alcoholic beer has emerged as a key segment within this trend, targeting consumers who seek to limit alcohol intake while preserving the characteristic flavor, aroma, and sensory complexity of conventional beer [3,4]. In addition to reduced ethanol content, non-alcoholic beer has been associated with potential functional properties, including antioxidant capacity and support of electrolyte balance during physical exercise [5]. The functional and sensory qualities of beer are strongly influenced by hops, which contribute to bitterness, aroma, microbial stability, foam quality, and flavor stability while serving as a major source of phenolic and antioxidant compounds. However, hop production is increasingly affected by climate change, resulting in reduced yields, lower α-acid concentrations, higher costs, and supply instability [6,7,8]. These challenges have intensified interest in identifying alternative or complementary sources of bitter and bioactive compounds that can support sustainable brewing practices without compromising product quality. Although alternative hop analogs have been studied in brewing research [9,10,11,12], most studies focus on primary plant materials and do not consider hop replacement within a zero-waste approach or the use of industrial by-products as functional brewing ingredients.

In parallel, clean-label and zero-waste concepts have become central drivers of innovation in the food and beverage sector. These approaches emphasize the use of natural ingredients and the valorization of food-processing by-products to reduce environmental impact while meeting consumer expectations for sustainability and transparency [13,14]. Within this framework, coffee-processing by-products have attracted increasing attention. Roasted coffee silverskin (RCSS), generated in large quantities during coffee roasting, represents a significant waste stream with limited high-value applications. Importantly, RCSS is rich in phenolic compounds, the composition of which varies depending on the coffee species from which it originates [15]. Previous studies indicate that the highest total phenolic content was found in Robusta coffee beans grown in India (168.27 mg GAE/100 g), followed by Arabica beans from the Rio-Minas region of Brazil (160.07 mg GAE/100 g) [16]. RCSS contains phenolic compounds such as chlorogenic, caffeic, and coumaric acids, along with tannins and other bitter constituents, highlighting its potential as a functional ingredient [17]. RCSS has been recognized as a potentially safe ingredient for food applications [18], and its use in food formulations has been explored in several studies. For example, it has been incorporated into yogurt [19], added to bakery products as a source of dietary fiber and prebiotics [20,21,22], and blended with instant coffee to create new coffee-based beverages [23]. While its application in beverages remains relatively underexplored, there are reports describing its use as a base for beverages and as an additive in smoothies [24,25]. Previous studies have demonstrated that the incorporation of RCSS positively influenced both physicochemical and sensory properties. Despite the growing interest in non-traditional brewing ingredients, including plant-based materials, the application of coffee-derived by-products in brewing remains insufficiently explored.

A total of 40 odorants were identified in RCSS, many of which have been reported as key odor-active compounds contributing to coffee flavor [26]. A comparison of volatile profiles between dried hops and RCSS revealed several shared compounds, including 1-octen-3-one (mushroom), 3-(methylthio)propanal (cooked potato), trans-4,5-epoxy-(E)-2-decenal (metallic), and α-humulene (balsamic) [26,27]. Linalool and related monoterpenes, which are responsible for floral–fruity notes, were also found in both hops and RCSS.

Despite these similarities, there is still a lack of systematic studies evaluating the use of RCSS as a source of bitter and phenolic compounds in non-alcoholic beer. In particular, its potential to partially replace hops while simultaneously enhancing the functional profile of the final product remains insufficiently explored.

Therefore, the aim of this study was to evaluate the feasibility of using RCSS as a functional ingredient in non-alcoholic beer production. The study focused on assessing the impact of RCSS addition on the phenolic content, antioxidant potential, and sensory characteristics of the resulting beers. This research represents a novel and application-oriented approach that integrates sustainability, clean-label principles, and functional product development, aligning with current industrial and market trends in brewing innovation.

## 2. Results and Discussion

### 2.1. Viable Counts of Total Microbes and Yeasts

Total microbes and yeasts [log CFU/mL] were analyzed in beer after the fermentation process and before the maturation of the beer. The results are shown in Table 1.

The total microbial counts ranged from 7.26 ± 0.45 to 7.63 ± 0.06 log CFU/mL across all analyzed samples. Similarly, the yeast counts ranged from 7.41 ± 0.10 to 7.60 ± 0.67 log CFU/mL. No statistically significant differences were observed among the used bitterness factor for either total microbial counts or yeast counts, as indicated by the identical letter notation. These results indicate that the use of different bitterness agents did not significantly affect the growth of the overall microbial population or yeasts during the fermentation period. Furthermore, the comparable levels of total microbes and yeasts suggest that no microbial contamination occurred during fermentation. This observation is particularly important, as microbial contamination is a common technological challenge in non-alcoholic and low-alcohol beers due to their reduced ethanol content, which provides weaker antimicrobial protection compared to conventional beers [28,29]. The absence of elevated total microbial counts beyond the yeast population indicates good microbiological stability and appropriate hygienic conditions during the fermentation process. This analysis is particularly important due to the lack of previous reports on the microbiological stability of beers produced with hop analogs.

### 2.2. Beer Analysis

Beers produced with different bitterness sources showed slight variations in pH, original gravity (OG), apparent extract (AE), apparent attenuation (AA), color (EBC), and protein concentration [g/L] (Table 2).

The pH values show statistical difference between the RCSS and control samples, meaning that the addition of RCSS significantly affected the pH value of the beer, reducing the acidity by 5%. This is a significant change in pH and would affect the beer’s sensory properties. The OG increased significantly among the samples supplemented with RCSS compared to the H. The OG were optimized to around 7°P. According to studies, an OG of 4.0 to 7.5°P is optimal for both arrested and limited fermentation processes [30]. Increasing the RCSS concentration in beer resulted in a statistically significant increase in AE among the samples. Apparent extract represents the residual dissolved solids remaining after fermentation and is influenced both by the initial wort composition and by the extent of sugar utilization by yeast [31]. The relationship between OG, AE, and the fermentation progress has been discussed in the classical brewing science literature as Balling’s attenuation theory, which is also used in the production of non-alcoholic beers [32]. No significant differences were observed in AA, which ranged from 7.41 ± 1.38% in H to 8.74 ± 0.29% in H+RSCC. These values indicate that fermentation proceeded normally in all formulations, and the addition of RCSS did not negatively affect yeast activity. Moreover, the AA value remained within a typical range for standard non-alcoholic beers and was consistent with previously reported values for wort and beer composition [33]. No statistically significant differences in AA or yeast counts were observed among the three beer samples, further suggesting that RCSS did not influence yeast metabolic activity. However, further research is needed to conclusively assess the effect of RCSS on yeast metabolism during fermentation, as well as its impact on aroma compound production.

The color of the beer was light yellow and became progressively more intense, shifting toward a distinct brown tint with increasing concentrations of RCSS. This effect is desirable, as consumers tend to prefer darker non-alcoholic beers and are often willing to pay more for such products, indicating that RCSS addition may enhance the market value of the final product [27]. Potential hop analogs are generated during the coffee roasting process, during which the Maillard reaction occurs. In the final stage of roasting, melanoidins are formed, which are responsible for the darker color of the beer [34]. In addition, the presence of melanoidins should be considered an added value, not only due to their antioxidant capacity but also because of their demonstrated prebiotic effects [35].

The alcohol content of all beer samples remained below 0.5%. Non-alcoholic beer typically refers to products with no or low alcohol content (≤0.5%), but its definition may vary by country [4]. The technological measures applied, namely the use of a specialized yeast strain, a low original extract, and controlled fermentation conditions, successfully resulted in the desired low alcohol content.

Protein, as one of the nutrients in beer, directly affects beer’s taste, color, and foam stability [36]. Around 80–85% of the proteins in beer originate from malt [37]. No statistically significant differences in protein content were observed among the three beer samples, indicating that the type of bittering agent used did not significantly affect the overall protein content of the final product.

**Figure 1:**
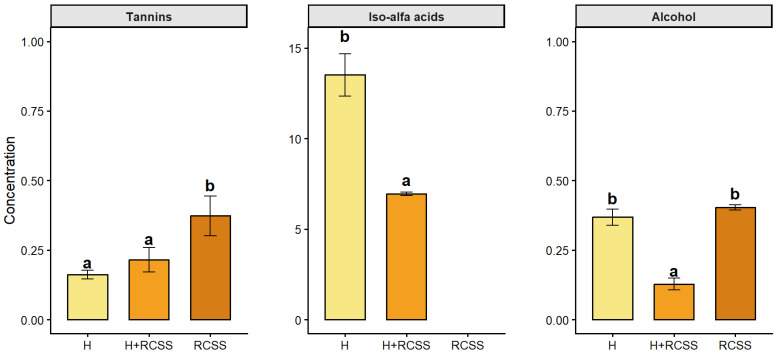
Tannin concentration [g/L] and iso-alfa acids [IBU] of beer samples H, H+RCSS, and RCSS. Results are expressed as mean ± standard deviation (n = 3). Different letters within the same chart indicate significant differences (*p* < 0.05).

The compounds responsible for bitterness are presented in Figure 1.

Beer bitterness is primarily attributed to iso-α-acids formed during wort boiling, while its intensity and quality are additionally modulated by non-hop resin compounds such as polyphenols [38,39]. With increasing concentrations of raw materials used to impart bitterness to beer, the tannin content increases significantly. The determination of iso-α-acid content can be compared only between samples H and H+RCSS, as these were the only samples containing hops. The iso-α-acid levels differ statistically between these samples, as they are dependent on the amount of hops used. RCSS is also characterized by the presence of tannins and melanoidins formed through the Maillard reaction during roasting [40]. Tannins contribute directly to major organoleptic properties, particularly taste attributes such as astringency and bitterness in RCSS [38]. In this study, tannins were estimated using the Folin–Ciocalteu assay, with tannic acid as the reference standard. It should be noted that this method is not specific for tannins and may also react with other phenolic compounds and reducing substances present in the sample. Therefore, the reported values should be interpreted as an estimate of tannin-related phenolics rather than the precise tannin content. Nonetheless, given the lack of published data on tannins in beers produced with RCSS, these findings may provide a useful reference for future research.

**Figure 2:**
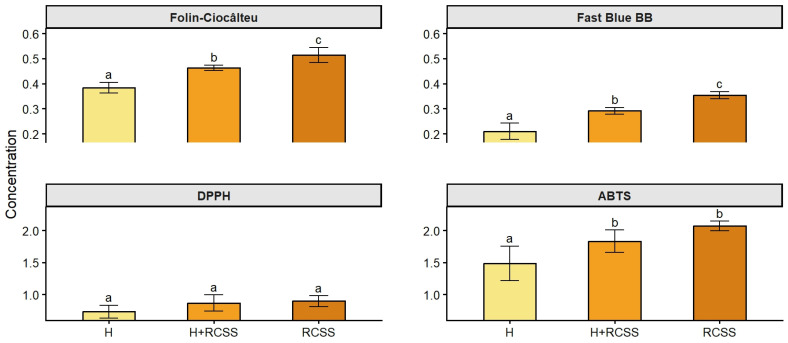
Total phenolic content and antioxidant capacity of beer samples H, H+RCSS, and RCSS determined by Folin–Ciocâlteu, Fast Blue BB, DPPH, and ABTS assays. Results are expressed as mean ± standard deviation (n = 3). Different letters within the same chart indicate significant differences (*p* < 0.05).

The results demonstrate clear differences in total phenolic content and the antioxidant capacity among the analyzed beer samples (Figure 2).

In both phenolic content assays, Folin–Ciocâlteu and Fast Blue BB, sample H exhibited the lowest values, where samples containing RCSS showed progressively significant higher concentrations. The correlation of these methods showed a high value of R^2^ = 0.9. The RCSS sample displayed the highest phenolic content using the Folin–Ciocâlteu method (0.51 ± 0.03 mg CGA/mL), indicating an enhanced contribution of phenolic compounds associated with the RCSS addition. A 34% increase in phenolic content was observed, as determined by this method, between the H beer and the RCSS beer. The FBBB assay proved to be the most suitable method for total phenolic content determination in teas and fruit juice drinks, as non-phenolic reducing compounds cause minimal interference. This method has not previously been used in beer analysis. Nevertheless, combining different assays can provide a more comprehensive evaluation, since each method responds to different antioxidant components [41]. The trend was observed for antioxidant capacity measured by the DPPH and ABTS assays. Sample H showed the lowest radical scavenging capacity, while H+RCSS and RCSS exhibited significantly higher antioxidant capacity. Notably, the ABTS assay revealed a pronounced increase in antioxidant capacity from H to H+RCSS and RCSS. The RCSS beer measured using ABTS assay showed nearly twofold higher values compared to the DPPH method. In the study by Rumpf et al. (2023), the antioxidant capacity of fruits, vegetables, and beverages measured using the ABTS assay was significantly higher than that measured with the DPPH assay [42]. The ABTS assay measures both hydrophilic and lipophilic antioxidants, whereas the DPPH assay is limited by radical solubility and reaction conditions [43]. Differences in reaction mechanisms and sensitivity also contribute to discrepancies between methods [42]. The consistent increase in values across all analytical methods indicates that the incorporation of RCSS substantially enhances both the phenolic content and antioxidant potential of the beer. The phenolic content and the antioxidant capacity of the control sample are comparable to the values previously reported in the literature for lager-style beers [44,45,46]. However, beers enriched with coffee, fruits, or food-processing by-products exhibit significantly higher values of phenolic content and antioxidant capacity compared to the control sample [46]. The use of RCSS as an additive in beer, and particularly its application as a bittering agent, has not yet been reported in the scientific literature.

These results confirm that RCSS addition leads to a measurable improvement in the bioactive properties of the beer, which may be associated with the increased presence of phenolic compounds known for their antioxidant capacity.

### 2.3. Sensory Evaluation

**Figure 3:**
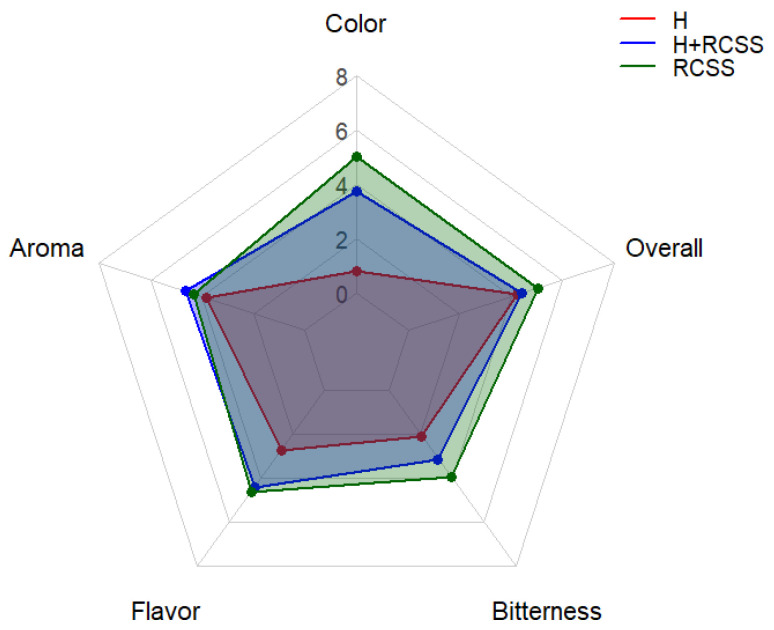
Sensory evaluation of beer samples H, H+RCSS, and RCSS.

Sensory evaluation of three beer varieties was performed, and results are presented in Figure 3.

The sensory evaluation showed differences among the H, H+RCSS, and RCSS samples across all assessed attributes. The H sample received the lowest scores, particularly for color and bitterness, while its aroma, flavor, and overall sensory quality were rated at moderate levels. The H+RCSS sample showed improved scores in all attributes compared to H, especially in color and bitterness. The RCSS sample achieved the highest ratings for most parameters, including color, bitterness, flavor, aroma, and overall sensory quality. Overall, the addition of RCSS enhanced the sensory quality of the samples.

Another study investigating the replacement of hops with carqueja [10] also reported, similar to RCSS, an increase in bitterness intensity after the addition of the hop analog. However, in that study, as well as in studies using artichoke [47] as a hop analog, the replacement resulted in lower overall sensory quality scores. In contrast, this effect was not observed in the case of RCSS, where overall sensory quality was maintained at a higher level.

**Figure 4:**
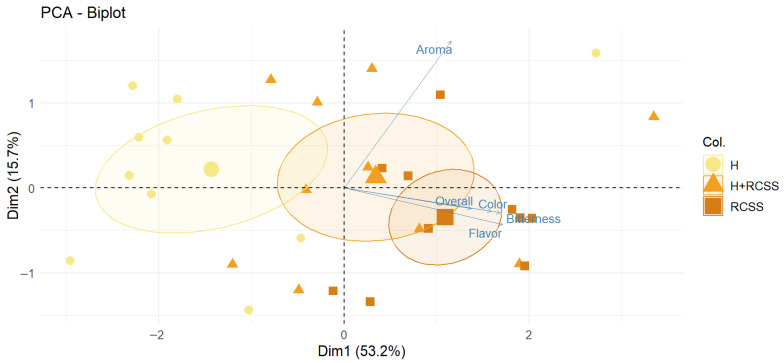
Principal Component Analysis (PCA) biplot showing the distribution of samples from three groups (H, H+RCSS, RCSS) in the space defined by the first two principal components (Dim1: 53.2%; Dim2: 15.7%). Different symbol shapes (circles, triangles, squares) represent the respective experimental groups. Colored ellipses indicate the confidence regions for each group. Arrows represent sensory variables (Aroma, Flavor, Bitterness, Overall Color), illustrating their contribution and direction in the PCA space. Partial overlap of the ellipses reflects similarities between groups and does not affect the scientific interpretation of the results.

The PCA biplot illustrates the multivariate relationships among sensory attributes and the differentiation of beer samples based on their sensory profiles (Figure 4).

The first principal component (Dim1), accounting for 52.5% of the total variance, primarily reflects differences in the intensity of bitterness, flavor, color, and global sensory perception, as evaluated by the trained panel. The second principal component (Dim2), explaining 15.8% of the variance, is mainly associated with variations in the sensory perception of aroma. A clear separation among the beer samples is observed along Dim1. Sample H is located on the negative side of Dim1, indicating lower intensity or a distinct sensory expression of bitterness and flavor attributes compared to the other samples. In contrast, samples H+RCSS and RCSS are positioned on the positive side of Dim1, reflecting higher intensity for these attributes.

Along the Dim2 axis, sample differentiation is mainly due to aroma evaluation, with samples showing varying levels of aroma intensity. The dispersion of points within each confidence ellipse represents interindividual variability among panelists. However, the relatively compact confidence areas indicate an acceptable level of panel agreement. Overall, the PCA results confirm systematic differences in the sensory profiles of the individual beer formulations. The addition of RCSS contributes to measurable and consistent modifications in the sensory attributes, particularly affecting bitterness, flavor, and aroma, as identified through expert sensory evaluation.

The RCSS sample shows the strongest association with bitterness and flavor, as indicated by its proximity to the corresponding vectors, suggesting a stronger contribution of these attributes to the overall sensory profile, which is consistent with the primary objective of the study to enhance sensory characteristics through the addition of RCSS. The bitterness provided by RCSS has a positive effect on the sensory profile. The source and concentration of bitter compounds from RCSS result in a bitterness quality that is more clearly perceived by trained panelists. Furthermore, RCSS addition led to the presence of additional aroma active compounds in the beer matrix [26]. The use of RCSS as a functional ingredient in food products has been associated with enhanced sensory properties, supporting its potential for broader food applications [25,48,49,50].

## 3. Materials and Methods

### 3.1. Beer Production

Pilsner malt (Viking Malt, Strzegom, Poland)was used to produce the wort. Multistage infusion mashing was carried out with two mashing ramps: 65 °C for 50 min and 73 °C for 10 min. Wort boiling with hops (Browin, Łódź, Poland) or finely ground RCSS (Café Roma, Piotrków Trybunalski, Poland) was carried out. For the standard recipe (H), 0.5 g/L of Lublin hops was used. For the mixed sample H+RCSS, 0.25 g/L of hops and 2.5 g/L of RCSS were used. The last sample RCSS was prepared using 5 g/L of RCSS. In all recipes, hops or RCSS was added at the beginning of boiling, which lasted 60 min. After boiling, the wort was quickly cooled to inoculation temperature. SafBrew™LA-01 dried yeast (*Saccharomyces cerevisiae* var. *chevalieri*) was inoculated at a rate of 60 g/hL. Fermentation was carried out for 48 h at 12 °C. This was followed by maturation for 14 days at 1 °C. All beer samples were prepared in triplicate. After the maturation period, the analyses were performed.

### 3.2. Number of Viable Total Microbes and Yeast

The viable microbial population in the beer samples was quantified using the plate count method after 4 days of fermentation. Briefly, 1 mL of each sample was aseptically diluted in 9 mL of sterile distilled water, followed by serial ten-fold dilutions. Appropriate dilutions were plated on selective solid media. Total microbial counts were determined on Plate Count Agar (PCA, Merck KGaA, Darmstadt, Germany) following incubation at 30 °C for 72 h. Yeasts were enumerated on Sabouraud Dextrose Agar (SDA, Merck KGaA, Darmstadt, Germany) supplemented with chloramphenicol (100 mg/L) and incubated at 30 °C for 72 h. The results are expressed as logarithmic values of colony-forming units per ml of sample (log CFU/mL).

### 3.3. Standard Analysis of Beer

The following physicochemical parameters were examined in this study: pH, original gravity, apparent extract, apparent attenuation, color, bitterness, and alcohol content, measured according to Sections 9.35, 9.4, 9.6, 9.8, and 9.2.1 of the European Brewery Convention methodology [51]. Protein was analyzed using Pierce™ Bradford Protein Assay Kit (Thermo Scientific™, Rockford, IL, USA). All analyses were performed in triplicate.

### 3.4. Phenolic Compounds and Antioxidant Capacity

The antioxidant capacity of the beer was measured using the DPPH assay (Sigma-Aldrich, St. Louis, MO, USA) described by Herald [52] and the ABTS assay (Sigma-Aldrich, St. Louis, MO, USA) proposed by Iriondo-DeHond [53]. The total phenolic content (TPC) of the beverages was determined using the Folin–Ciocâlteu (Supelco, Bellefonte, PA, USA) method described by Ainsworth [54] and the Fast Blue BB (MedChemExpress, Monmouth Junction, NJ, USA)method described by Medina [55], with chlorogenic acid (Sigma-Aldrich, St. Louis, MO, USA) as the standard. Results were expressed as mg of chlorogenic acid per ml of beer. All analyses were performed in triplicate.

### 3.5. Tannins

Tannins were estimated using Folin–Ciocâlteu reagent [56]. About 0.1 mL of the beer was added to a volumetric flask (10 mL) containing 7.5 mL of distilled water, 0.5 mL of Folin–Ciocâlteu reagent, and 1 mL of 35% sodium carbonate (Sigma-Aldrich, St. Louis, MO, USA) solution and was then diluted to 10 mL with distilled water. The mixture was shaken well and kept at room temperature for 30 min. Absorbance for the test and standard solutions was measured at 700 nm. Estimation of the total tannin content was carried out in triplicate and expressed as g/L of tannic acid (Sigma-Aldrich, St. Louis, MO, USA) in the sample.

### 3.6. Sensory Analysis

The sensory panel consisted of 10 members from the Institute of Fermentation Technology and Microbiology, Lodz University of Technology. All panelists were experienced and had previously participated in sensory evaluations of beer and other fermented beverages. The panel training was conducted in eight 2 h sessions in accordance with ISO 8586:2012 [57]. During these sessions, assessors were trained using beers and a variety of fermented beverages. Immediately prior to the sensory evaluation, the cooled beer samples were carbonated. Panelists were served 50 mL of each sample at a temperature of 12 °C, poured without foam. The samples were coded with three-digit random numbers and presented in randomized order to avoid bias. The panel evaluated the intensity of selected sensory attributes, including aroma, flavor, and color, as well as overall sensory quality, using a structured ten point intensity scale (10 = extremely intense; 1 = not perceptible). Each sample was evaluated twice by each assessor.

### 3.7. Statistical Analysis

Statistical analysis was performed using one-way ANOVA followed by Tukey’s HSD post hoc test to determine significant differences between beer samples (H, H+RCSS, and RCSS). Results are expressed as mean ± SD, and differences were considered significant at *p* < 0.05. Different letters indicate statistically significant differences between groups. All statistical analyses were performed using RStudio (version 2025.05.1, Posit Software, PBC, Boston, MA, USA).

## 4. Conclusions

This study demonstrates for the first time the potential for using RCSS in beer production. The addition of RCSS provided bitterness that was acceptable to panelists while increasing the total phenolic content and antioxidant capacity of the beer. Importantly, RCSS had no negative effect on the fermentation process. The proposed use of this coffee by-product promotes cost optimization and is consistent with zero-waste and sustainability strategies. However, comprehensive studies are needed to assess the long-term stability of beers brewed with RCSS. Since the present research focused on a preliminary set of three formulations, determining the optimal RCSS dosage and clarifying its technological effects and sensory impact on the final product will be valuable. Additionally, the identification of volatile flavor compounds and quantitative profiling of individual polyphenols will provide further useful insights.

## Figures and Tables

**Table 1:** Viable counts of total microbes and yeasts in beer samples H, H+RCSS, and RCSS after 4 days of fermentation.

	Total Microbes	Yeasts
	[log CFU/mL]	
H	7.63 ± 0.06 ^a^	7.58 ± 0.27 ^a^
H+RCSS	7.26 ± 0.45 ^a^	7.41 ± 0.10 ^a^
RCSS	7.49 ± 0.85 ^a^	7.60 ± 0.67 ^a^

Results are expressed as mean ± standard deviation (n = 3). Different letters within the same column indicate significant differences (*p* < 0.05).

**Table 2:** The original gravity [OG], apparent extract [AE], apparent attenuation [AA], pH, color and protein concentration of beer samples H, H+RCSS and RCSS.

	OG [°P]	AE [°P]	AA [%]	pH [-]	Color [EBC Units]	Protein [g/L]
H	6.70 ± 0.10 ^a^	6.20 ± 0.01 ^a^	7.40 ± 1.38 ^a^	5.28 ± 0.03 ^a^	4.65 ± 0.11 ^a^	3.22 ± 0.13 ^a^
H+RCSS	7.02 ± 0.03 ^b^	6.40 ± 0.01 ^b^	8.74 ± 0.29 ^a^	5.14 ± 0.05 ^b^	11.74 ± 0.32 ^b^	3.15 ± 0.12 ^a^
RCSS	7.11 ± 0.01 ^b^	6.52 ± 0.03 ^c^	8.30 ± 0.50 ^a^	5.02 ± 0.07 ^b^	18.02 ± 0.13 ^c^	3.10 ± 0.11 ^a^

Results are expressed as mean ± standard deviation (n = 3). Different letters within the same column indicate significant differences (*p* < 0.05).

## Data Availability

The original contributions presented in this study are included in the article. Further inquiries can be directed to the corresponding author.

## Abbreviations

The following abbreviations are used in this manuscript:

RCSS	Roasted Coffee Silverskin
OG	Original Gravity
AE	Apparent Extract
AA	Apparent Attenuation
